# Craniotomy Simulator with Force Myography and Machine Learning-Based Skills Assessment

**DOI:** 10.3390/bioengineering10040465

**Published:** 2023-04-12

**Authors:** Ramandeep Singh, Anoop Kant Godiyal, Parikshith Chavakula, Ashish Suri

**Affiliations:** 1 Neuro-Engineering Lab, Department of Neurosurgery, All India Institute of Medical Sciences, New Delhi 110029, India; 2 Department of Physical Medicine and Rehabilitation, All India Institute of Medical Sciences, New Delhi 110029, India

**Keywords:** surgical skills, drilling, bone matrix, 3D printing, force myography, artificial intelligence

## Abstract

Craniotomy is a fundamental component of neurosurgery that involves the removal of the skull bone flap. Simulation-based training of craniotomy is an efficient method to develop competent skills outside the operating room. Traditionally, an expert surgeon evaluates the surgical skills using rating scales, but this method is subjective, time-consuming, and tedious. Accordingly, the objective of the present study was to develop an anatomically accurate craniotomy simulator with realistic haptic feedback and objective evaluation of surgical skills. A CT scan segmentation-based craniotomy simulator with two bone flaps for drilling task was developed using 3D printed bone matrix material. Force myography (FMG) and machine learning were used to automatically evaluate the surgical skills. Twenty-two neurosurgeons participated in this study, including novices (n = 8), intermediates (n = 8), and experts (n = 6), and they performed the defined drilling experiments. They provided feedback on the effectiveness of the simulator using a Likert scale questionnaire on a scale ranging from 1 to 10. The data acquired from the FMG band was used to classify the surgical expertise into novice, intermediate and expert categories. The study employed naïve Bayes, linear discriminant (LDA), support vector machine (SVM), and decision tree (DT) classifiers with leave one out cross-validation. The neurosurgeons’ feedback indicates that the developed simulator was found to be an effective tool to hone drilling skills. In addition, the bone matrix material provided good value in terms of haptic feedback (average score 7.1). For FMG-data-based skills evaluation, we achieved maximum accuracy using the naïve Bayes classifier (90.0 ± 14.8%). DT had a classification accuracy of 86.22 ± 20.8%, LDA had an accuracy of 81.9 ± 23.6%, and SVM had an accuracy of 76.7 ± 32.9%. The findings of this study indicate that materials with comparable biomechanical properties to those of real tissues are more effective for surgical simulation. In addition, force myography and machine learning provide objective and automated assessment of surgical drilling skills.

## 1. Introduction

Craniotomy is a surgical procedure in which a flap of the skull bone is removed in order to expose the dura and access the brain [1]. It is an integral part of all neurosurgical procedures and involves the usage of high-speed drills and other specialized instruments [2]. During a craniotomy procedure, dural tear or rupture can have several adverse consequences, such as cerebrospinal fluid (CSF) leak, infection, hematoma, or brain herniation [3,4]. Furthermore, patients suffering from traumatic head injuries in rural areas require immediate surgical intervention even in the absence of neurosurgeons [5]. Therefore, competent training of craniotomy procedures in a safe and repeatable environment is essential for neurosurgeons as well as community general surgeons [6,7]. However, the typical time-based apprenticeship paradigm for craniotomy training lacks hands-on experience in the early stages and may compromise patient safety later on. Therefore, the concept of competency-based training is being adopted widely [8]. This method includes use of simulation-based training models to hone skills outside the operating room in a safe and repeatable manner [9].

Physical as well as virtual reality simulators can be used to provide simulation-based training [10,11]. Among these, physical simulators provide real-time haptic feedback and are more effective for psychomotor skills training [10]. Patient-specific physical simulators constructed using computed tomography (CT) and magnetic resonance imaging (MRI) data of patients provide a more realistic training environment [12]. Typically, 3D printing is employed to fabricate the intricate structures of a physical simulator due to their complex shape [13]. Various 3D printed materials, such as acrylonitrile butadiene styrene (ABS) plastic, polyamide, gypsum, and polymers are used to fabricate cranial bones for surgical simulators [14]. However, the majority of these lack biomechanical properties and do not accurately replicate natural bone drilling [15]. Realistic tissue fidelity is essential to provide a tool–tissue interaction feel similar to surgery. Therefore, physical neurosurgical simulators require materials that mimic the biomechanical properties of the skull [16].

Evaluation of surgical skills is essential for competency-based training, since it provides trainees with feedback on their performance [17]. Traditionally, the evaluation of surgical skills relied on an expert surgeon assessing the performance of trainees; however, this method suffers from inter-observer bias and the limited availability of experts [18]. The virtual reality simulators have a defined workspace, which makes skill validation considerably more straightforward [19]. However, with physical simulators, evaluating surgical skills is more challenging. The objective evaluation on physical simulators can be carried out using computer vision techniques or by using electronic sensors. Video-based analysis includes tracking and analysis of the surgical instrument movements [20]. However, video cannot capture the data related to tool–tissue interaction, such as applied force, muscular workload, cognitive workload, and gaze pattern. Therefore, various sensors, including electroencephalography (EEG) [21], electromyography (EMG) [22], inertial measurement units (IMU) [23], force sensors [24], and eye-tracking [25], have been employed in previous studies to evaluate surgical skills. However, the sensor-based data collection system must be minimal and should not impede the natural movements of the surgeons [26]. 

The aim of the present study was to develop a craniotomy simulator and system for the objective evaluation of surgical skills. The specific contributions of the present work are as follows: (i) The study described the development and validation of a realistic neurosurgical craniotomy simulator for microscopic drilling activity; (ii) 3D printed materials that mimic biomechanical properties were used to fabricate skull and dura; (iii) a force myography band was developed to measure the variation in forearm muscle radial force patterns during surgical drilling activity; (iv) various machine learning algorithms were used to classify the level of surgical expertise.

## 2. Methodology

### 2.1. Simulator Design and Fabrication

**Figure 1:**
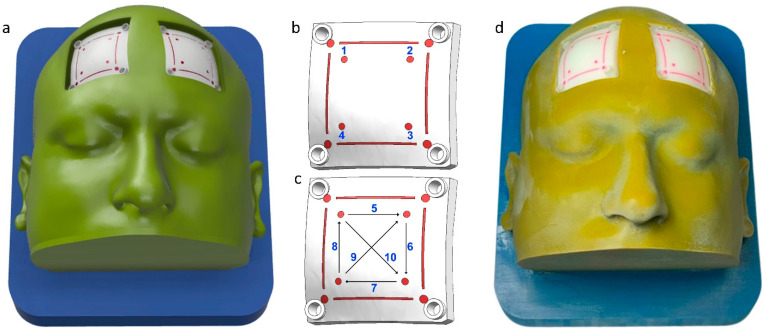
Craniotomy simulator with removable flaps for drilling activity. (**a**) CAD model, (**b**) 1–4 burr hole drilling activity, (**c**) 5–8 straight lines, and 9–10 diagonal line drilling activity, and (**d**) 3D printed prototype.

A patient’s CT scan data were obtained after written consent. The data were used to segment the scalp, skull, and dura using Simpleware ScanIP software R-2021.03 (Synopsys Inc, Mountain View, CA, United States). The editable 3D models of these anatomical structures were exported in the STEP format using the ScanIP NURBS module. The STEP files were imported into the NX 2007 (Siemens, Munich, Germany) computer-aided design software. Two square-shaped cutouts were created in the frontal region of the scalp to expose the skull. Inside these cutouts, two flaps for the craniotomy training were created. Support structures were modeled around these two flaps and holes were made to fix the flaps to the scalp. An angulated base plate was designed to provide surgical position for the craniotomy procedure. Burr holes and lines were also designed on the drilling patches to provide markings for defined drilling activity [27]. The drilling activity includes making four burr holes, four linear lines, and two diagonal lines. A digital anatomy 3D printer (J750, Stratasys, Rehovot, Israel) was utilized to fabricate the components of the simulator. The outer body and base plate were fabricated using a combination of Vero cyan, magenta, and yellow materials. The drilling patches were fabricated using bone matrix and dura in Agilus materials, respectively [16]. The CAD model, drilling activity, and 3D printed prototype of the craniotomy simulator are shown in Figure 1a–d, respectively. 

### 2.2. Force Myography Band

**Figure 2:**
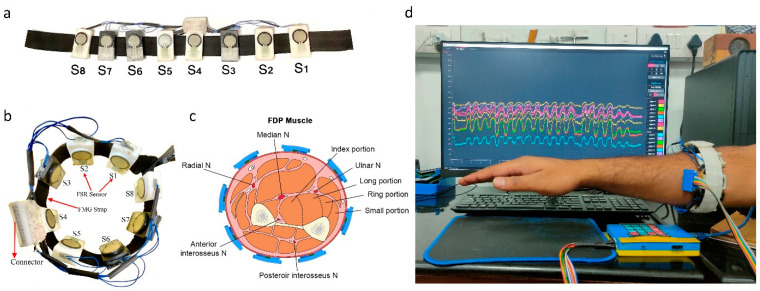
Data collection setup. (**a**) Force myography band using an array of eight FSR sensors, 3D printed mounts, and elastic strap, (**b**) FMG band sensor placement sites on the forearm, (**c**) forearm cut section depicting sensor placement sites with respect to muscles, and (**d**) FMG band donned on the forearm and data recording.

For neurosurgical drilling, small forces are applied on the bone using brush-like strokes of the drill bit. Evaluation of tool–tissue contact forces can, therefore, provide insights of surgical competency. As it was difficult and non-ergonomic to place force sensors between a tool and a surgeon’s hand, force myography (FMG) was used to evaluate the applied forces. FMG measures the volumetric changes in the arm muscles via radial force and has been widely used for hand gesture prediction [28]. Accordingly, an FMG band with eight flexible force sensitive resistors (FSR) was designed as a wearable force-sensing device (FSR 400, sensitivity 0.2–20 N, Interlink Electronics Inc., Camarillo, CA, United States). The FSR sensors were calibrated using a universal testing machine (UTM) (H5KS, SDL Atlas, Rock Hill, SC, USA). The sensors were fixed on the custom-made 3D printed mounts (40 × 22 mm) with hard ABS plastic material to support the back filament of FSR. The design of the FSR mount allowed for the passage of wiring and an elastic strap. The average circumference range of the band was kept at 22 cm non-stretched, but can be extended to 28 cm after stretching. The data collection setup is depicted in Figure 2.

A custom-made printed circuit board (PCB) was used to capture the signal of eight FSR sensors of the FMG band. The PCB consists of a Arduino mega 2560 microcontroller (Arduino, Budapest, Hungary), Bluetooth module (HC-05), and Battery monitoring system (3S 20A Li-ion Lithium Battery, 18650 Charger PCB BMS Protection Board 12.6 V Cell) to provide power to the system and receive data at a remote desktop. A voltage divider circuit was used to capture the variation in the sensitivity of the FSR sensors and tune them to operate in the desired curve. A constant voltage of 5 volts was supplied at one terminal of the FSR, and the other terminal was connected to the analog input pin of the microcontroller. The same analog pin was connected with a 10 kΩ resistor. To receive and visualize the signal, a freeware serial terminal application (IDE processing, version 3.5.3) was used. IDE is an open-source platform to receive the signal serially and store it in the .txt or .csv formats.

### 2.3. Data Collection and Experimental Protocol

**Figure 3:**
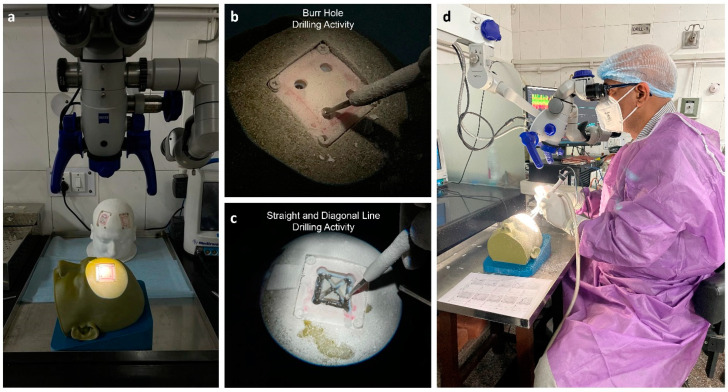
Simulation-based training using a craniotomy simulator. (**a**) Operating microscope and craniotomy simulator, (**b**) burr hole drilling activity, (**c**) straight- and diagonal-line drilling activity, and (**d**) experimental setup.

Twenty-two subjects were recruited for this study after giving written consent. All subjects were male and right-handed. Among these, 6 were experienced neurosurgeons (age: 49 ± 3.5 years) with more than 10 years of experience, 8 were senior residents (age: 29 ± 1.4 years) with 3 years experience, and the other 8 were junior residents (age: 26 ± 1 years) with no experience of craniotomy procedures. The study was approved by the institute’s ethics committee (IEC-206/9 April 2021). The FMG strap was donned on the dominant hand (forearm) of the participants. The seventh sensor was positioned at the line between the styloid process of the ulna and the medial epicondyle of the humerus. The remaining sensors were evenly distributed along the forearm’s diameter. This configuration of sensors enabled comparable forearm muscle radial force measurements among different participants. The participants were advised to perform three different activities, i.e., (1) drilling four burr holes, (2) drilling four linear lines, and (3) drilling two diagonal lines. All participants performed the defined drilling task on the patches placed on the right side of the simulator. An encoder (grove rotary angle sensor) was attached on the foot pedal of the drilling machine to identify the drill on and off position based on the threshold values. During the drilling trials, eight-channel FMG data were acquired using the experimental setup depicted in Figure 3.

### 2.4. Simulator Validation 

The effectiveness of the craniotomy simulator was evaluated by conducting a survey among all participants, including expert neurosurgeons, senior residents, and junior residents. After performing the training experiment with the simulator, the participants provided feedback on parameters including usability, haptics, comfort, and suitability. Usability refers to the simulator’s usefulness for effective skill development outside the operating room. The haptic feedback evaluates the degree of resemblance between 3D printed bone surrogate and real bone drilling. The comfort relates to the ease of performing the drilling activity while donning the FMG band on the forearm. Suitability refers to the neurosurgeons’ recommendation to include the developed simulator in the residency program. All participants rated their experience on a scale ranging from 1 to 10, with 1–3 indicating some value, 4–6 indicating good value, and 7–10 indicating excellent value. The neurosurgeons’ scores were tabulated in a Microsoft Excel spreadsheet. 

### 2.5. FMG Data Preprocessing

**Figure 4:**
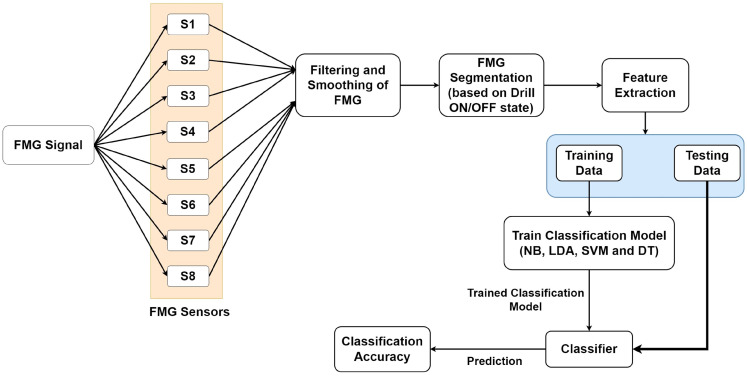
Methodology for the analysis of force myography data (FMG) data for skills evaluation.

Participants’ eight-channel FMG data were imported into MATLAB R2020b for further analysis. As reported in the literature, a typical human hand movement frequency is less than 4.5 Hz [29]. Therefore, the collected FMG data of each participant was filtered using the low pass Butterworth filter of the fourth order with a cut-off frequency of 10 Hz. In addition, a 10-point moving average filter was used to further smoothen the FMG data. The complete FMG data of each participant was segmented into drilling and resting state based on the threshold value of the foot potentiometer marker. The segmented data were then forwarded to the feature extraction step. The complete methodology for FMG data analysis is shown in Figure 4. 

### 2.6. Feature Extraction 

Feature extraction of the FMG signals was based on the earlier literature [30,31]. The simplified and computationally less complex features were used in the present study. Based on the preliminary analysis, five features were selected, as depicted in Table 1.

### 2.7. Classification

**Figure 5:**
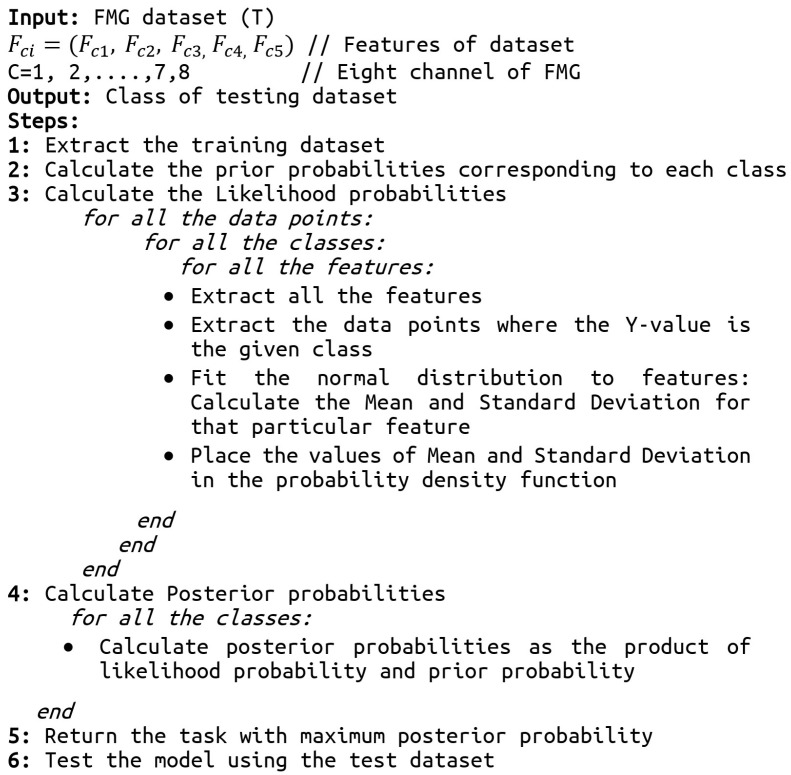
The pseudo-code of the skills assessment using the naïve Bayes algorithm.

In this study, we evaluated the performance of naïve Bayes, linear discriminant analysis (LDA), support vector machine (SVM), and decision tree classifiers for classification of surgical skills into expert, intermediate, and novice categories. The naïve Bayes (NB) classifier is a subcategory of the Bayes classifier [32]. It is a supervised machine learning algorithm that is based on a probabilistic classification approach. It is best suited for the high dimensional dataset with the assumption that the predictors are independent of each other. The pseudo-code for skills assessment using FMG data with the naïve Bayes algorithm is given in Figure 5. LDA is a classifier which is flexible, powerful, and has low computational cost [33]. By examining the linear combinations of input variables, it separates the assigned classes. SVM classifiers are capable of learning from smaller amounts of data [34]. The implementation of SVM involves creating a hyperplane as a decision surface to maximize the distance between a positive and a negative example. It performs on a higher-level feature space which is created by nonlinearly transforming the n-dimensional input vector into a K-dimensional feature space. The decision tree is a classifier with tree structure, and is a powerful tool for classification [35]. It is composed of inner and leaf nodes, which represent decision thresholds and predictions, respectively. Comparison of extracted features with each inner node of the decision tree, from the root node to the leaf node, is the typical process of classification. 

### 2.8. Evaluation

As the present study contains a limited dataset of 22 participants, we employed the leave one subject out cross-validation for the training and testing of the classifier. In this procedure, data from one of the subjects was used as the testing dataset, while the data of the rest of the subjects were used as the training dataset. This process was repeated until all subjects’ data were used once as a testing dataset. The performance parameters including accuracy, precision, recall, and f1 score were calculated using the following equations:

(1) $$Accuracy=\frac{\left(TP+TN\right)}{(TP+TN+FP+FN}$$

(2) $$Precision=\frac{TP}{\left(TP+FP\right)}$$

(3) $$Recall=\frac{TP}{\left(TP+FN\right)}$$

(4) $$F1\;Score=\frac{2*Precision*Recall}{\left(Precision+Recall\right)}$$

For a better understanding of the classification performance, we have calculated the confusion matrix. The diagonal values of the confusion matrix show the correctly classified subjects between the actual class and estimated class.

### 2.9. Statistical Analysis

Due to the non-parametric nature of the data, we utilized the Kruskal–Wallis test with Dunn’s multiple comparison test to compare the classification accuracy of various classifiers. The significance level was considered to be 0.05.

## 3. Results

### 3.1. Simulator Validation by Neurosurgeons

**Figure 6:**
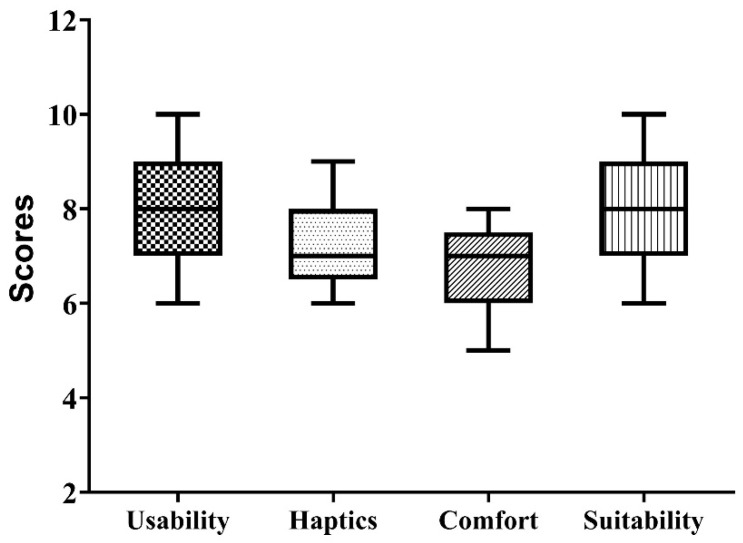
Result of neurosurgeon’s survey-based evaluation of the developed craniotomy simulator.

The results of the validation survey by neurosurgeons were compared by plotting boxplots, as shown in Figure 6. The results show that the vast majority of participants strongly felt that the craniotomy simulator was easy to use and had excellent value (average score = 8.0) for skill building outside the operating room. Regarding the haptic properties of the 3D printed bone matrix material, the material was found to have a good value (average score = 7.1) for use in surgical drilling simulation. The comfort of performing the activity while wearing the FSR band was also found to have a good value (average score = 6.7), and the participants reported no ergonomic discomfort while wearing the FMG band. The developed simulator was found to have excellent value (average score = 7.9) for inclusion in the neurosurgery residency program. 

### 3.2. Classification

The performance of four different classifiers used in the present study was analyzed using accuracy, precision, recall, and F1 score. The classification accuracy was highest for the naïve Bayes classifier (90.0 ± 14.8), followed by the decision tree classifier (86.2 ± 20.8) and linear discriminant classifier (81.9 ± 23.6). The least performing classifier was the support vector machine classifier (76.7 ± 32.9). The precision, recall and F1 score also followed the pattern of the classification accuracy, as depicted in Table 2. 

**Figure 7:**
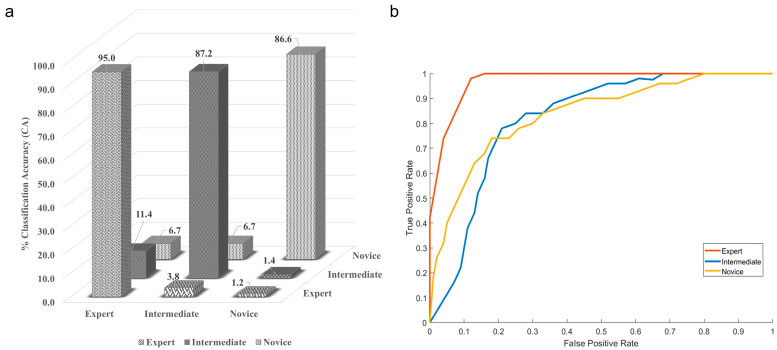
(**a**) Confusion matrix, (**b**) ROC curve for the highest performing naïve Bayes classifier.

The results of the Kruskal–Wallis test with Dunn’s multiple comparison test showed that there was no statistically significant difference (*p* = 0.6) between the accuracy of different classifiers. However, the maximum mean classification accuracy, precision, and recall with lesser standard deviation were achieved using the naïve Bayes (NB) classifier. This shows that the naïve Bayes classifier was the best classifier for categorizing neurosurgeons into expert, intermediate, and novice categories. Figure 7a shows the confusion matrix for the naïve Bayes classifier. 

Here, the diagonal elements show the correctly classified class, while the non-diagonal shows the misclassification. The experts are 95% correctly classified as the experts, and the NB classifier misclassified experts as intermediate 3.8% of the time and as novices 1.2% of the time. The classification accuracy for correctly classifying intermediates was 87.2, while the NB classifier misclassified intermediate as experts 11.4% of the time and as novices 1.4% of the time. The novices are correctly classified 86.6% of the time, and the misclassification equally divides between the expert and novice at 6.7%. The graphical representation of the naïve Bayes classifier is also shown with the help of the ROC curve in Figure 7b. The confusion matrix and ROC curve indicate that the classifier is majorly confused between the expert and the intermediate class.

## 4. Discussion

Adequate resident training requires hands-on experience, but operative neurosurgery affords few such chances [36]. Moreover, the pressure of performing well, time constraints, and the fear of mistakes hinder adequate learning [37]. It may also lead to an erroneous evaluation of the residents’ surgical aptitude on the part of the supervisor [38]. Simulation systems offer a unique solution for resident training in a safe environment as well as their unbiased evaluation [39]. In the present study, a craniotomy simulator was developed for teaching high-speed drilling skills. In addition, an FMG band was developed for objective and automated evaluation of surgical skills using machine learning.

The cranial bone is a complex structure consisting of external outer layers of compact, high density cortical bone and an inner layer consisting of a low density, irregular porous structure [40]. The surgeons experience a unique haptic sensation when bone dust is produced by surgical drilling [41]. In the past decade, a variety of materials have been employed to fabricate cranial bones for surgical simulators, as detailed in Table 3. However, these materials lack haptic feedback comparable to cranial bones. Therefore, we used the recently introduced bone matrix material of the Digital Anatomy printer (J750, Stratasys, Rehovot, Israel) to fabricate skull bone. The results of the neurosurgeon evaluation indicate that this material provided a comparable surgical drilling feel. Since high-speed surgical drilling generates heat, continuous irrigation is necessary to dissipate the heat [42]. However, with bone matrix material, the use of irrigation caused reduced drilling efficiency. Consequently, continuous drilling during the experiment caused the drill bit to become extremely hot, and a piece of moistened gauze was used to cool it in between. In addition, the lack of irrigation caused bone dust to spread across the drilling site, which was cleared by using suction. Therefore, further improvements in the bone material are required for effective drilling simulation.

The evaluation of the surgical skills and competence is a challenging task even in simulation models. Different scoring methods, such as OSATS, are widely used and are validated assessment scales. Using these scales, an expert surgeon evaluates and scores the performance of trainee surgeons. However, such an evaluation unavoidably increases human bias and places an excessive load on experts, making adaptation and generalization difficult on a larger scale. Therefore, automatic evaluation of surgical skills is important to provide feedback to trainees and to reduce the burden of expert evaluators. Different wearable sensors have been used in various studies for automatic objective evaluation of surgical skills, as depicted in Table 4. However, the sensor-based data collection system must be compact and cause minimal disruption to the natural movements of the surgeons. Therefore, we collected forearm muscle radial force data using an FMG band for skills evaluation. Neurosurgeons determined that the band, which was worn on the forearm, did not interfere with the surgical training experiments. The accuracy achieved using the present system was on par with the existing sensors used for the assessment of surgical skills.

The data collected from the FMG band was used to classify the surgical expertise using machine learning algorithms. The naïve Bayes classifier achieved the highest classification accuracy. During the study, we found that out of 22 subjects, the classifier was able to achieve acceptable classification accuracy for 19 subjects but, for 3 subjects, the classifier demonstrated a minimum classification accuracy of 60%, and this accuracy decreased even further for the other 3 classifiers. The classification accuracy, therefore, displays a considerable standard deviation. If these three participants are excluded from the study, the mean classification accuracy increases from 90 to 95%, and the standard deviation decreases from 14.8 to 9%. Therefore, we might consider these individuals to be non-conventional neurosurgeons with a unique approach of performing the high-speed drilling-based procedure. The present study can be extended by including the data of a large number of participants. Moreover, simulators for other surgical tasks, such as suturing, cutting, incising, and tumor resection can be developed using similar methods, and the potential of FMG bands or other electronic sensors for surgical skills evaluation can be analyzed.

## 5. Conclusions

It is concluded that the anatomically accurate surgical simulator with materials having comparable haptic properties to real tissue has great potential for use in surgical training. The study participants opined that the bone matrix material used to fabricate cranial bones provided comparable haptic feedback for surgical drilling. Objective assessment of surgical skills provides trainees with immediate feedback on their performance and reduces the burden on expert evaluators. The FMG band developed in the present study was found to be a simple and cost-effective solution for machine learning-based automated evaluation of surgical drilling skills. 

## Figures and Tables

**Table 1:** Features extracted from the eight-channel FMG data.

S. No.	Features	Description
1.	$F_{C1}=mean\left(X_{C}\right)\;\;\;\;\;\;\;\;\;\;\;\;\;\;\;\;\;\;\;\;\;\;\;\;\;\;\;\;\;\;$	Average value of FMG signal
2.	$F_{C2}=Var\left(X_{C}\right)\;\;\;\;\;\;\;\;\;\;\;\;\;\;\;\;\;\;\;\;\;\;\;\;\;\;\;\;\;$	Variance of FMG channel
3.	$F_{C3}=WLen\left(X_{C}\right)$	Cumulative length of the FMG signal
4.	$F_{C4}=MedFreq\left(X_{C}\right)$	Median frequency dividing the total power of the signal into two equal halves
5.	$F_{C5}=CoV\left(X_{C}\right)=\frac{SD\left(X_{C}\right)}{Mean(X_{C})}\times 100$	Coefficient of variation in the FMG channel

Note: $F_{Ci}$ (*i* = 1,2,3,4,5) stands for the features of the FMG signal ($X_{C}$). The subscript ‘C’ (c = 1,2,3….8) stands for the eight channels of the FMG signal. $X_{C}$ is a N-length FMG data vector of the specific activity of an individual. Concatenating all the features extracted from all the FMG channels, a 40-dimensional feature vector is generated (5 features × 8 channels) for a particular activity.

**Table 2:** Performance metrics for the four different classifiers [Mean (SD)].

Classifier	Accuracy	Precision	Recall	F1 Score
Naïve	90.0 (14.8)	89.6 (4.7)	90.8 (5.1)	90.0 (1.5)
LDA	81.9 (23.6)	82.2 (4.2)	82.6 (11.0)	82.3 (7.1)
SVM	76.7 (32.9)	76.6 (0.9)	77.2 (8.4)	76.8 (3.6)
DT	86.2 (20.8)	86.1 (3.0)	87.5 (9.2)	86.6 (5.0)

**Table 3:** Materials used for fabrication of cranial bones for surgical simulation.

S. No.	Author	Cranial Bone	Fabrication Methods
1.	Present Study	Bone matrix	3D printing—polyjet
2.	M. Lai et al. (2021) [43], R.G. Nagassa et al. (2019) [44], J.R. Ryan et al. (2016) [45]	Gypsum powder	Stereolithography 3D printing
3.	Q. Lan et al. (2020) [46]	Photosensitive polymers	Polyjet 3D printing
4.	M. Licci et al. (2020) [47]	Polylactic acid (PLA)	Fused deposition modeling 3D printing
5.	C.L. Craven et al. (2018) [48]	Thickened polyurethane resin	3D printing and casting
6.	K.W. Eastwood et al. (2018) [49]	VisiJet C4 Spectrum plastic material	ColorJet 3D printing
7.	T. Mashiko et al. (2017) [50]	ZP130 Powder	ColorJet 3D printing
8.	J. Muto et al. (2017) [51]	Polamide nylon (30% to 90%) and glass beads (10% to 70%)	Selective laser sintering 3D printing
9.	D.R. Cleary et al. (2017) [52]	ABS plastic	Fused deposition modeling 3D printing
10.	K. Kondo et al. (2015) [53]	Plaster (zp150 powder and zb63 clear binder)	Binder jetting 3D printing
11.	G. Coelho et al. (2015) [54]	Fiberglass	Molding
12.	D. Inoue et al. (2013) [55]	Acrylic plastic	Polyjet 3D printing

**Table 4:** Wearable sensors used for objective evaluation of surgical skills.

S. No.	Authors	Surgical Task	Sensor Used	Body Location	Results
1.	Present Study	Drilling	Force myography band	Forearm	90% classification accuracy using naïve Bayes
2.	T. Manabe et al. (2022) [21]	FLS (pegboard transfer and suturing)	Electroencephalography (32–channel)	Head	90% classification accuracy using regularized micro-state-based CSP (common spatial pattern)
3.	A. Zulbaran-Rojas et al. (2021) [22]	Graft Anastomosis	Inertial measurement unit and electromyography (flexible sensors)	Dorsum of each hand and flexor digitorium of the dominant hand	Significant correlation between mean OSATS score and sensor parameters
4.	K. Evans-Harvey et al. (2020) [56]	Dissection of Calot’s triangle	Eye gaze tracking	Eyeglass frame	Correlation between laparoscopic screen dwell time and OSATS scoring [r = 0.655, *p* < 0.05]
6.	K.F. Kowalewski et al. (2019) [57]	Suturing, knot tying	Myo-armband	Forearm	MAE OSATS score: 3.7 ± 0.6
7.	L. Sbernini et al. (2018) [58]	Single interrupted suture and simple running suture	Sensory glove with flex and inertial sensors	Hand	Median classification error rate of 0.61% for interrupted and of 0.57% for running sutures using ANN
8.	M. Uemura et al. (2018) [59]	Suturing on rubber sheet	Six-degree-of-freedom magnetic tracking sensor	Tip of surgical instrument (needle holder)	79% classification between expert and novice surgeons using neural network
9.	H. Rafii-Tari et al. (2015) [60]	Endovascular Catheterization	Force/torque sensor	Aortic arch phantom base plate	Classification accuracies of 94% (expert) and 98% (novice)
10.	K. Harada et al. (2015) [61]	Anastomosis on artificialblood vessels (0.7-mm)	Infrared optical motion tracking markers, aninertial measurement unit, and strain gauges	Tool/needle end	Expert surgeons performed in a shorter time, shorter tool path, and with less force during needleextraction
11.	I.H. Suh et al. (2015) [62]	Laparoscopic surgery	Electromyography	Forearm and hand muscles	Significant distraction effects for EMG measures (EMGenv, *p* < 0.004; EMGfmed, *p* = 0.031).
13.	I. Oropesa et al. (2014) [63]	Grasp, Pull and transfer tasks	TrEndo tracking system	Surgical instrument	Mean classification accuracy of 71% (LDA), 78.2% (SVM), and 71.7% (ANFIS)

## Data Availability

The data are not publicly available due to privacy restrictions.
